# Long lasting impregnated mosquito net (LLIN) utilisation, incidence of fever and therapeutic itineraries: the case of Mifi health district in western Cameroon

**Published:** 2017-11-01

**Authors:** Patrick P. Nkamedjie, Ghyslaine B. Dongho, Rodrigue B. Mabvouna, Gianluca Russo, Martin S. Sobze

**Affiliations:** 1 Institute for Research, Socio-economic Development and Communication (IRESCO), Yaoundé, Cameroon; 2 Department of Public Health and Infectious Diseases, “Sapienza” University of Rome, Italy; 3 Department of Biology, Faculty of Medicine and Surgery, University of Rome “Tor Vergata”, Rome, Italy; 4 Department of Biomedical Sciences, University of Dschang, Cameroon

## Abstract

**Background:**

Long Lasting Impregnated mosquito Net (LLIN) use is effective against malaria in endemic tropical areas. However, its utilisation remains limited. Among the most common clinical signs of malaria is fever and many studies have reported the existence of different local ways of handling fever; amongst which uncontrolled used of antimalarial drugs. We investigated LLINs use and its impact on fever outcomes and the various therapeutic measures used to deal with fever episodes.

**Materials and methods:**

Data was extracted from a cross sectional descriptive and analytic study performed between January and April 2014 in Mifi health district. Data was collected in households through a face to face interview with standard household questionnaires, treated and analysed using Epi Info statistical software version 3.5.3.

**Results:**

A total of 317 participants were interviewed with average age 33.2 years (SD = 10.8). Female respondents were predominant (85.2%; n=270). Most participants attended secondary education (53.6%; n= 170). Married marital status was most represented (58.1%; n= 185). 75.4% (n=239) of households owned at least 1 LLIN against an estimated average district coverage of 1 LLIN for 3.3 persons. Average bednet usage for households owning at least 1 LLIN was 57.9%. Utilisation of LLINs in households reduced fever episodes by 5.3%, (p=0.36). To handle fever episodes, road side medicines represented priority therapeutic itinerary for most of our respondents (95.0%; n=301).

**Conclusions:**

Although LLINs are effective in reducing fever episodes, their utilisation remains low. Self-medication to treat fever seems to be prominent. There is a need to optimise communication for behavioural change strategies to promote consistent LLIN use and anti-malarial therapy, assisted by qualified health personnel.

## 1 Introduction

Malaria remains one of the most widespread diseases affecting human race in tropical and sub-tropical regions of the world [1]. Transmission occurs in all six WHO regions. Globally, an estimated 3.3 billion people are at risk of being infected. Of this total, 1.2 billion were at high risk (>1 case per 1000 population), 47% of them were living in Africa while 37% came from southeast Asia [2]. According to the World Health Organization (WHO), 214 million cases of malaria occurred in 2015, leading to 438,000 deaths. Eighty -eight percent of these deaths occurred in the African region [3]. The disease constitutes over 10 % of Africa’s overall disease burden, accounting for 40% of public health expenditure, 30–50% of in-patient hospital admissions and up to 50% of out-patient visits in endemic areas [4]. Over the last century new efforts have been made to control malaria. Among these advances is the use of insecticide-treated nets (ITNs), now mostly long-lasting insecticide-treated nets (LLINs). LLINs are known to kill mosquitoes and have repellent properties that reduce the number of mosquitoes that enter the house or the net (e.g. through holes) [4]. They are estimated to be twice as effective as untreated nets [5] and offer greater than 70 % protection compared to no nets [6].

Over the past decade, malaria incidence has fallen by at least 50% in one-third of the countries where the disease is endemic. These gains have been made through a combination of interventions, including timely diagnosis and treatment using reliable tests and anti-malarial drugs; indoor spraying with safe insecticides; and the use of LLINs to protect people from mosquito bites at night [7].

Mosquito net ownership is far from universal despite the aforementioned gains. Ownership rates remain low in many malarious regions or amongst particular risk groups in endemic settings. Furthermore, net ownership in itself is not synonymous with net utilisation. Individuals who own (or who have available) mosquito nets must use them in order for the potential health impact to be fully realised [8].

Fever, one of the main clinical signs of malaria, may have different etiologies, but in malaria endemic areas clinicians should always exclude a *Plasmodium* infection as cause of fever. Also, in malaria-endemic countries, self-medication is still widespread [9,10], potentially contributing to an increased spread of drug resistance [11]. To the best of our knowledge, although few data exist on bednet ownership and usage in the Cameroon West Region [12], data concerning these health indicators in the Mifi Health district are absent. The present study aimed to assess LLIN use, its impact on self-reported fever episodes and household management of presumptive malaria based on febrile syndromes in the Mifi Health District, west Cameroon.

## 2 Materials and methods

### 2.1 Study design, area and sampling

The present cross sectional descriptive and analytic study was conducted between January and April 2014. Predominantly urban, the district had an estimated population of 266 988 residing in 20 health areas with children below 5 accounting for 16% of the overall population [13]. Mifi health district is located 1100 m above sea level, the climate is equatorial and characterised by a long rainy season (9 months) and a dry season [13].

Health areas were grouped into rural and urban and randomly selected proportionally to the number in each category. Three out of eight urban and 4/12 rural health areas were sampled and the number of households to be visited determined according to the total population of the health area. Overall, 317 households were surveyed in the 7 selected health areas.

### 2.2 Data collection and analysis

Data was collected in the randomly selected households using a standard pretested questionnaire during a face-to-face interview with consented participants. Questions were related to LLINs utilisation, febrile diseases incidence and their management at household level. We estimated LLIN coverage per household and then multiplied the values by 10 to have whole numbers. Determination of LLINs usage was done by getting the proportion of household inhabitants (including houses without LLINs) who slept under the net the night preceding the interview. Analysis of data was done using EPI Info statistical software version 3.5.3 (CDC, Atlanta, GA, USA). Self-reported cases of fever episodes was obtained by asking respondent on its outcome during the previous 3 months. Descriptive statistics of mean, standard deviation, and frequency distribution was used to summarise data. We used linear regression with significance level set at P < 0.05 to test the association between LLIN utilisation and fever outcome.

### 2.3 Ethical considerations

This study was approved by the Department of Biomedical Science (University of Dschang) and the Mifi District Health Services. All participants were adults and the questionnaires were administered only after obtaining informed consent. Data obtained was managed in order to strictly respect the participant’s confidentiality.

## 3 Results

The acceptance rate for participation in the study was very high, 97.5% (n=317/325). As summarised in Table 1, participants were mainly females (85.8%; n=270), with a good educational level (secondary school or University) (61.8%; n=196); and the majority was married (58.1%; n=184).

**Table 1:** Socio-demographic characteristics (n=317).

Variable	n	%
Gender		
Male	47	14.8
Female	270	85.2
Educational level		
None	27	8. 5
Primary	94	29.8
Secondary	170	53.6
University	26	8.1
Marital status		
Single	44	14.0
Cohabitation	54	17.1
Married	184	58.1
Separated	6	1.8
Divorced	3	0.9
Widow	26	8.1
Occupation		
Trader	45	14.2
Farmer	49	15.5
Government worker	11	3.5
Dress maker	32	10.1
House wife	109	34.4
Barber/hair maker	10	3.2
Other	61	19.2

LLIN coverage was estimated per tens of household, per person and per zone. Overall, 75.5% (n=239/317) of visited households had at least 1 LLIN, while others had more than one net. Coverage was not significantly (P=0.35) higher in rural settings (19 LLINs for 10 households) compared to urban settings (17 LLINs for 10 households). Average district coverage was estimated to be 1 LLIN for every 3.3 persons. The most recent mass LLINs distribution campaign was undertaken three years earlier, in 2011 [12]. Average LLIN usage in the district for people living in homes having at least 1 LLIN (n=239) was 57.9%. Data also revealed no significant difference (P=0.08) between LLINs usage in urban (58.57%) and rural (57.03%) settings.

Fever was defined as any person having body temperatures above normal (37 degrees), measured using a thermometer or confirmed by health personnel. With regards to the National Malaria Control Programme, all suspected malaria cases must be confirmed using a Rapid Diagnostic Test (RDT) and simple malaria cases should be managed with Artemisinin Combine therapy (ACT) both within the community and in health facilities [13]. However, we did not find data to support these indicators in the sampled health areas.

Table 2 details the outcome of fever the previous 3 months before interview day; per health area category. In general, in households where inhabitants consistently used LLINs, the incidence of fever registered the previous 3 months appeared to be 5.3% (P-value=0.36) on average lesser compared to houses where LLINs are not used. Our findings also reveal that most people suffering from fever (48.41%) were children age 0 to 5 five years.

**Table 2:** Incidence of fever in households (n=317) in the 3 months preceding the interview.

Health area category	Fever?	Proportion (%)	Number of households
Urban	Yes	54.78	64
	No	45.22	55
Rural	Yes	61.6	122
	No	38.4	76

Following these fever episodes, most of the respondents (95.0%) tended to street vendors for treatment. Modern pharmacies other than health facilities, home treatment and rushing to a health facility represented the priority therapeutic itinerary in 78%, 65% and 13.6% of households respectively. No data was collected regarding the drugs purchased to manage fever.

## 4 Discussion

Our study reports how LLIN usage influences fever episode outcome and the various approaches undertaken by community members in response to fever episodes. For LLINs to be effective in reducing malaria incidence, its coverage and consistent use by the targeted populations must equal at least 80% [14,15]. 75 % Of the households visited owned at least 1 LLIN, which is close to what was observed (70.9%) in a survey done in Jimma Zone, southwestern Ethiopia [16]. Despite the fact that acceptable coverage according to WHO is 1 LLIN for every 1.8 inhabitants [17], it appears that this indicator is far below this norm (1 LLINs for 3.3 persons) in Mifi health district. This indicator in lower compared to the suggestions made following an investigation in Democratic Republic of Congo, where coverage was 1 LLIN for every 2.5 persons [18]. Actual LLIN utilisation was also low since only 57.9% of people living in homes with at least 1 LLIN acknowledged that they slept under their nets the night before the interview. This is higher than what was reported in a study carried out by Birhanu *et al.* [16], who reported usage as low as 38.4%. However, this finding seems low compared to the findings of Ntuku *et al.* (68.3%) following a survey carried out in Kasaï Occidental Province, Democratic Republic of Congo [18]. Malaria is an acute febrile illness [19] and populations refer to malaria as ‘fever’ or ‘strong fever’ [20]. Therefore, by acting as a barrier between the mosquito vector and the human host, bednets can significantly reduce the incidence of fever in a community [20]. The non-significant nature of this indicator in our finding could be due to a sample size. Many authors have highlighted self-medication (i.e., participants procure and administer antimalarials on their own without medical advice or buy them from the roadside drug vendors or quacks) as a prominent issue regarding fever and malaria management [9,10]. Similarly, a considerable proportion of the participants (95.0%) practiced self-medication and the main reason for this was limited financial means to attend to a health facility for appropriate diagnosis and management. Nsagha *et al.*, in a survey conducted in a semi-urban area of Cameroon, reported much lower levels of self-medication (55.7%) [21].

## 5 Conclusion

Consistent use of LLINs has shown to be effective in reducing fever and malaria incidences. However, in the Mifi health district, coverage and utilisation remain below recommended targets. Self-medication to cure fever and suspected malaria cases seems to be an alarming issue. There is need to channel more efforts toward increasing LLIN coverage and promotion of its consistent use. Attention also need to be focused towards raising awareness on the dangers of self-medication which is a major source of drug resistance.
